# Soft Tissue Substitutes in Periodontal and Peri-Implant Soft Tissue Augmentation: A Systematic Review

**DOI:** 10.3390/ma17051221

**Published:** 2024-03-06

**Authors:** Roberto Rotundo, Gian Luca Pancrazi, Alessia Grassi, Lara Ceresoli, Giovanna Laura Di Domenico, Vanessa Bonafede

**Affiliations:** 1 Periodontology Unit, University Vita-Salute and IRCCS San Raffaele, 20132 Milan, Italy; 2 Oral Surgery, University Vita-Salute and IRCCS San Raffaele, 20132 Milan, Italy

**Keywords:** biomaterials, gingival recession, connective tissue graft, free gingival graft, soft tissue substitutes, keratinized tissue, dental implants

## Abstract

Background: Different extracellular matrix (ECM)-based technologies in periodontal and peri-implant soft tissue augmentation have been proposed in the market. The present review compared the efficacy of soft tissue substitutes (STSs) and autogenous free gingival grafts (FGGs) or connective tissue grafts (CTGs) in mucogingival procedures to increase keratinized tissue (KT) width around teeth and implants. Methods: Two independent examiners performed an electronic search on MEDLINE and the Cochrane Library based on the following PICOS format: (P) adult patients; (I) soft tissue substitutes and FGGs/CTGs; (C) STSs vs. CTGs; STSs vs. FGGs; STSs vs control; (O) KT width gain; (S) systematic reviews, randomized controlled trials. Studies published before November 2023 were included. Results: Around teeth, all biomaterials showed superior performance compared to a coronally advanced flap (CAF) alone for treating gingival recessions. However, when compared to CTGs, acellular dermal matrices (ADMs) yield the most similar outcomes to the gold standard (CTGs), even though in multiple recessions, CTGs continue to be considered the most favorable approach. The use of STSs (acellular matrix or tissue-engineered) in combination with apically positioned flaps (APF) resulted in significantly less gain in KT width compared to that achieved with FGGs and APFs. Around dental implants, free gingival grafts were deemed more effective than soft tissue substitutes in enhancing keratinized mucosa width. Conclusions: Based on the available evidence, questions remain about the alternative use of soft tissue substitutes for conventional grafting procedures using free gingival grafts or connective tissue grafts around teeth and implants.

## 1. Introduction

Since 1963, the autogenous free gingival graft (FGG) has been used in periodontal surgery to enhance the width of the attached gingiva around teeth [1,2]. The same technique was also used to cover exposed dental roots [3]. Later on, in order to achieve better esthetic results, complete root coverage, increased keratinized tissue width (KTW), and long-term treatment stability, an autogenous subepithelial connective tissue graft (SCTG) combined with a coronally advanced flap (CAF) has been largely used and, nowadays, is recognized as the surgical technique with the highest performance [4]. This approach is, therefore, acknowledged as the “gold standard” for treating both single and multiple gingival recession defects (GRDs) around teeth and dental implants [5].

Despite its high success rate, the SCTG shows some drawbacks, including the necessity for two surgical sites (recipient and donor), pain and discomfort during donor site healing, and limited availability of donor tissue [6].

Meanwhile, several studies have started to test the use of soft tissue substitutes (STSs) in mucogingival surgery, revealing interesting results, mainly based on their easier and less invasive approach, eliminating the need to harvest tissue from the palate [4,7,8]. Since the 1980s, STSs have been introduced as alternative materials to autogenous grafts for increasing gingival width, showing their advantages over autogenous grafts, such as their widespread availability, avoidance of a secondary surgical site, reduced surgical time, and patient preference. It should be considered that the risk of moderate/severe postoperative swelling and pain increases by 3% and 4%, respectively, for each minute of the surgical procedure [9,10]. Therefore, the reduction in surgical time represents one of the most determinant factors in patient morbidity.

Scaffolds, based on their origin, have been categorized as allogenic, xenogeneic, alloplastic, and living constructs (including cells). The most commonly used biomaterials include acellular dermal matrix grafts, a single/bilayer collagen matrix, a volume-stable collagen matrix, a porcine-derived acellular dermal matrix, and polymeric matrices of human amniotic membranes [11].

The present review compared the efficacy of soft tissue substitutes (STSs) and autogenous free gingival grafts (FGGs) or connective tissue grafts (CTGs) in mucogingival procedures for soft tissue augmentation around teeth and implants.

## 2. Soft Tissue Substitutes

Biomaterials used as connective tissue substitutes should ideally show certain properties, such as ease of adaptation and positioning at the level of the affected site, stabilization of the blood clot, integration with host tissues, and reduction in time and pain related to the removal of an autologous graft. The most commonly used STSs are described as follows.

### 2.1. Dermal Matrix

This is a soft tissue graft obtained from human or porcine dermis that undergoes a decellularization process. It is used not only in dentistry but also in aesthetic medicine and surgery. In dentistry, the purpose of its use is to avoid a second surgical site due to a graft harvest, and it is applied in root coverage procedures, as well as in augmentation of periodontal and peri-implant soft tissues [4,12].

### 2.2. Human Amniotic Membrane

This biomaterial is obtained from healthy donors during cesarean sections and is composed of the membrane that covers the amniotic cavity, which undergoes a process of preparation and consequent elimination of the cellular component, maintaining a single epithelial layer, the basement membrane, and collagen. It also contains growth factors that contribute to the properties of this biomaterial in terms of healing and angiogenesis [13].

### 2.3. Porcine-Derived Collagen Matrix

A collagen matrix of porcine origin is a dense and smooth material used to promote adhesion to the recipient site, angiogenesis, and tissue integration. Like any scaffold, it supports clot stabilization and provides stability. It is widely used in dentistry, particularly in periodontal surgery, soft tissue augmentation, and the treatment of gingival recessions. Within the porcine-derived matrices, it is possible to identify a bilayer collagen matrix or a volume-stable collagen matrix. The latter shows, as a main characteristic, maintenance of good stability, elasticity, and volume. It stimulates angiogenesis, fibroblast growth, and tissue integration. Unlike the bilayer collagen matrix, which can also be used in an open environment, the volume-stable matrix requires submerged healing [4].

### 2.4. Polymeric Matrices

There are also polymeric matrices on the market derived from proteins, polysaccharides, and polynucleotides. One of the main advantages of natural polymers is biocompatibility. They stimulate healing and act as scaffolds for tissue regeneration. The main derivative protein is collagen, although one of the main disadvantages of collagen derivatives seems to be tissue contraction. Depending on the characteristics required, biomedical engineering with the combination of molecules can achieve various types of synthetic scaffolds, which, compared to natural derivatives, have the advantage of longer shelf life, as well as showing greater elasticity and tensile strength, compensated however by lower biodegradability, so they are often used in combination with natural polymers [14].

## 3. Materials and Methods

This systematic review was conducted based on the following question, “What is the efficacy of soft tissue substitutes in mucogingival surgery around teeth or around dental implants in terms of soft tissue augmentation and root coverage?” This question was designed according to the format of the following PICOS strategy [15]:

- Population (P): adults (≥18 years) presenting reduced keratinized tissue around teeth and implants,
- Intervention (I): root coverage procedures, soft tissue augmentations,
- Comparisons (C): soft tissue substitutes vs. no treatment or connective tissue graft/free gingival graft,
- Outcomes (O): keratinized tissue width, soft tissue thickness, root/implant coverage with more than 6 months of follow-up,
- Study design (S): systematic reviews, randomized clinical trials

### 3.1. Inclusion Criteria

Studies published before November 2023, written in English, were included. Systematic reviews and randomized controlled studies dealing with the use of collagen-based soft tissue substitutes in periodontal plastic surgery (soft tissue augmentation procedures) around teeth and implants with more than 6-month follow-up were selected.

### 3.2. Exclusion Criteria

In vitro or in vivo animal studies, retrospective clinical trials, clinical trials without control, case reports, narrative review articles, editorials, opinion pieces, surveys, conferences, and commentary articles were excluded. Studies on bio-modulators or non-collagen-based matrices were excluded, as well as studies without a full text available and studies not written in English.

### 3.3. Outcomes

Primary outcomes: Root coverage; gingival/peri-implant soft tissue augmentation.

### 3.4. Strategy Search

The search process was performed by two different reviewers using different electronic databases: MEDLINE (via PubMed), Scopus, and the Web of Science. The search strategy was based on the MeSH terms in PubMed and adapted to each database. The following search queries were adopted: (“collagen matrix” [All Fields] OR “soft tissues substitute” [All Fields] OR “Biocompatible Materials/therapeutic use” [Mesh terms] OR “Collagen Type I/therapeutic use” [Mesh terms] OR “Collagen Type III/therapeutic use” [Mesh terms] OR “human fibroblast-derived dermal substitute” [All Fields] OR “acellular dermal matrix” [All fields] OR “dermal matrix allograft” [All Fields] OR “soft tissue allograft” [All Fields] OR “xenogeneic collagen matrix” [All Fields] OR “Mucograft” [Supplementary Concept] OR “Alloderm” [Supplementary Concept] OR “Fibro gides” [All Fields] OR “Mucoderm” [Supplementary Concept] OR “Novomatrix” [All Fields] OR “Derma” [All Fields] OR “connective tissue graft” [All fields] OR “connective tissue” [Mesh Terms] OR “subepithelial connective tissue” [All fields] OR “periodontal and plastic surgery” [All fields] OR “soft tissue graft” [Mesh Terms] OR “coronally advanced flap” [All fields] OR “Bilaminar technique” [All fields] OR “Free Gengival Graft” [All fields] OR “Dental Implants” [Mesh Terms] OR “Tooth Root/surgery” [Mesh terms] OR “Gingivoplasty/methods” [Mesh terms]) AND (“gingival recession” [All fields] OR “reduced keratinized tissue” [All fields] OR “gingival recessions” [Mesh Terms] OR “gingival recession treatment” [All fields] OR “gingival recession coverage” [Mesh Terms] OR “recession near gingiva” [All fields] OR “recession defect” [Mesh Terms] OR “exposure near root” [All fields] OR “exposed near root” [Mesh Terms] OR “gingiva near defect” [All fields]) AND (“root coverage” [All fields] OR “keratinized tissue width” [All fields])) NOT (Comment [Publication Type] OR Congress [Publication Type] OR Editorial [Publication Type] OR Case Reports [Publication Type] OR Clinical Conference [Publication Type] OR Comment [Publication Type] OR Consensus Development Conference [Publication Type]).

### 3.5. Selection of Studies, Data Extraction and Synthesis

After removing duplicate records, titles, and abstracts (when available) of all the reports identified through both electronic and manual searches, they were independently screened by two review authors (GLP and VB). When studies met the inclusion criteria or when insufficient data from abstracts for evaluating inclusion criteria were available, the full article was obtained. The full text of all the eligible studies was independently assessed by the two review authors (GLP and VB). All the studies matching the inclusion criteria then underwent data extraction. Any disagreements were resolved through discussion or consultation with other authors (RR, GLDD).

## 4. Results

**Figure 1:**
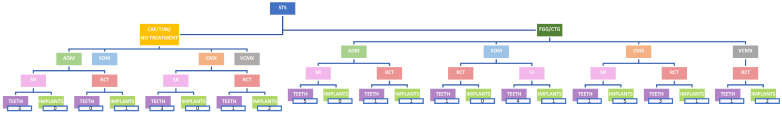
Flow chart diagram of selected studies. For each soft tissue substitute (STS), the related number of systematic reviews (SR) and randomized controlled trials (RCT) are reported. ADM: allogeneic dermal matrix; CAF: coronally advanced flap; CTG: connective tissue graft; FGG: free gingival graft; TUN: tunnel technique; XDM: xenogeneic dermal matrix; CMX: xenogeneic collagen matrix; VCMX: volume-stable collagen matrix.

The literature search flow diagram is shown in Figure 1. Out of 789 articles identified through the electronic search, following the removal of duplicates, 17 records were selected based on titles, abstracts, and full-text assessment (see Table 1, Table 2, Table 3 and Table 4).

In Figure 1, some included articles have been cited more than once since they deal with different treatments considered in the present review [4,16,17,18,19,20,21,22].

**Table 1:** Soft Tissue Substitutes vs. No Treatment around Teeth.

Study Type	Authors	Year	Surgical Procedure	Test Group	Control Group	No. of Patients/ No. of Teeth or Implants	Follow-Up (Months)	CRC	mRC	KTW	STT	Conclusion
RCT [23]	Jepsen et al. [23]	2017	CAF for single recessions	CAF + CMX	CAF	18/36	36	T: 61.1% C: 38.9%	T: 91.7 ± 12.05% C: 82.77 ± 17.03%	T: from 2.14 ± 1.21 mm to 4.06 ± 1.55 mm C: from 2.22 ± 1.39 mm to 3.25 ± 0.81 mm	T: from 0.93 ± 0.27 mm to 1.52 ± 0.41 mm C: from 0.96 ± 0.34 mm to 1.11 ± 0.41	In CAF + CMX group, mRC, CRC, KTW, and STT showed better outcomes than CAF alone
SR [4]	Chambrone et al.	2018	1. CAF for multiple recessions 2. CAF for single recession	1. CAF + ADM2. CAF + ADMG	1. CAF 2. CAF	48 studies in total, 2 studies evaluated	>6	from 0% to 91.6% for ADMG from 7.7% to 81.8% for CAF	from 50% to 96% for ADMG from 55.9% to 95.4% for CAF	NA	NA	ADMG appears as the soft tissue substitute that may provide the most similar outcomes to those achieved by SCTG
SR [21]	Moraschini et al.	2020		1. CAF + CMX 2. CAF + CMX 3. CAF + CMX 4. CAF + ADM	1. CAF 2. CAF 3. CAF 4. CAF	27 studies in total, 2 studies evaluated	12	NA	C: 81.4% ± 23.4 T: 93.2% ± 10 C: 75% ± 26.2 T: 76.2% ± 28 C: 75% ± 30 T: 87% ± 19 C: 74.9% ± 28.0 T: 94.8%	C: 0.7 ± 1.04 mm T: 1.07 ± 0.87 mm C: 0.64 ± 1.16 mm T: 1.05 ± 1.19 mm C: 1.1 ± 1.3 mm T: 0.6 ± 1.7 mm C: 0.60 ± 0.36 mm T: 1.21 ± 0.23 mm	NA	Biomaterials increase the effectiveness of RC in comparison with CAF alone. ADM demonstrated the best results

ADM: allogeneic dermal matrix; C: control group; CAF: coronally advanced flap; CRC: complete root coverage; mRC: mean root coverage; NA: not applicable; STT: soft tissues thickness; T: test group; CMX: xenogeneic collagen matrix.

**Table 2:** Soft Tissue Substitutes vs. No Treatment around Dental implants.

Study Type	Authors	Year	Surgical Procedure	Test Group	Control Group	No. of Patients/ No. of Teeth or Implants	Follow-Up (Months)	CRC	mRC	KTW	STT	Conclusion
RCT [18]	Frizzera et al.	2018	STA (Immediate implant placement and provisionalization)	STA + CMX	No soft tissue augmentation	16/16	12	T: NA C: NA	T: NA C: NA	T: NA T: NA C: NA	T: from 0.98 to 2.1 mm C: from 1 to 2.11 mm	CMX reduced MPR, provided better contour of the alveolar ridge, and increased STT
RCT [19]	Zuiderveld et al.	2018	STA (in conjunction with implant placement)	STA + CMX	No soft tissue augmentation	40/40	12	T: NA C: NA	T: loss of 0.17 ± 1.3 mm C: loss of 0.48 ± 1.5 mm	T: NA C: NA	T: NA C: NA	CMX does not result in a more favorable esthetic outcome than when no soft tissue graft was applied
RCT [20]	Lee et al.	2023	STA in conjunction with implant placement	STA + ADM	No soft tissue augmentation	31/31	12	ADM maintained buccal soft tissue contours 3–5 mm below the initial soft tissue margin	T: NA C: NA	Changes were not significantly different between the groups	T: from 1.34 ± 0.25mm to 2.57 ± 0.30 mm C: from 1.18 ± 0.31 mm to 1.18 ± 0.31 mm	STA enhanced STT and maintained soft tissue contours but did not prevent peri-implant mucosal recession

ADM: allogeneic dermal matrix; C: control group; CRC: complete root coverage; mRC: mean root coverage; NA: not applicable; STA: soft tissues augmentation procedure; STT: soft tissues thickness; T: test group; CMX: xenogeneic collagen matrix.

**Table 3:** Soft Tissue Substitutes vs. Free Gingival Graft/Connective Tissue Graft around Teeth.

Study Type	Authors	Year	Surgical Procedure	Test Group	Control Group	No. of Patients/ No. of Teeth or Implants	Follow-Up (Months)	CRC	mRC	KTW	STT	Conclusion
RCT [24]	Aroca et al.	2013	TUN for multiple recessions	MCAT + CMX	MCAT + CTG	22/156	12	T: 42% C: 85%	T: 71 ± 21% C: 90 ± 18%	T: from 2.1 ± 0.9 mm to 2.4 ± 0.7 mm C: from 2.0 ± 0.7 mm to 2.7 ± 0.8 mm	T: from 0.8 ± 0.2 mm to 1.0 ± 0.3 mm C: from 0.8 ± 0.3 mm to 1.3 ± 0.4 mm	CMX reduce surgical time and patient morbidity but give lower CRC when used in conjunction with MCAT
SR [4]	Chambrone et al.	2018	1. CAF for single recession 2. CAF for multiple recessions 3. CAF for single recession	1. CAF + ADMG 2. CAF + CMX 3. CAF + ADMG	1. CAF + CTG 2. CAF + CTG 3. CAF + CTG	48 studies in total, 3 studies evaluated	>6	from 0% to 91.6% for ADMG from 18.1% to 95.6% for SCTG	from 50% to 96% for ADMG from 64.7% to 99.3% for SCTG	NA	NA	There was insufficient evidence of a difference in GR reduction and KTW gain between ADMG + CAF and SCTG + CAF
SR [16]	de Carvalho Formiga et al.	2020	CAF	1. ADM 2. ADM 3. XDM 4. CMX	1. CTG 2. CTG 3. CTG 4. CTG	14 studies in total, 4 studies evaluated (conducted after 2010)	>6	No statistically significant differences	The CTG increased the MRC (+7.6 percentage points)	On 2 mm recessions, CTG showed superiority above other biomaterials, but on 3 mm recessions, it seemed to have the same results	NA	CTG, acellular dermal matrix allograft and xenogenic collagen matrix provided similar results for root coverage
RCT [25]	McGuire et al.	2021	VP	VP + CMX	VP + FGG	23/	6-8 y	T: NA C: NA	T: −0.07 ± 1.26 C: −0.17 ± 0.78	T: −0.09 ± 1.30 C: 0.20 ± 0.72	T: NA C: NA	Recession levels were maintained equivalently by both therapies
RCT [26]	Tonetti et al.	2021	CAF for multiple recessions	CAF + CMX	CAF + CTG	125/307	36	T: 3% C: 3%	T: NA C: NA	C: from 2.8 ± 1.3 mm to 0.5 ± 1.0 mm T: from 2.6 ± 1.2 mm to 0.0 ± 1.2 mm	T: NA C: NA	CMX reported shorter time to recovery, lower morbidity, and a more natural appearance of tissue texture and contour
RCT [27]	Elmahdi et al.	2022	TUN for multiple recessions	MCAT + ADM	MCAT + CTG	12/69	9	T: from 2.87 ± 0.31mm to 0.76 ± 0.65 mm C: from 2.76 ± 0.89mm to 0.53 ± 0.48 mm	T: NA C: NA	T: from 3.03 ± 0.72mm to 3.12 ± 0.69 mm C: from 2.65 ± 0.92 mm to 3.82 ± 1.3 mm	T: from 1.10 ± 0.20 mm to 1.65 ± 0.39 mm C: from 1.33 ± 0.54 mm to 2.26 ± 0.63 mm	The use of ADM may represent a valid alternative to SCTG when used in conjunction with MCAT
RCT [28]	Molnár et al.	2022	TUN for multiple recessions	MCAT + XDM	MCAT + CTG	22/114	9 years	T: 1% C: 1%	T: 23.07 ± 44.5% C: 39.7 ± 35.17%	T: from 2.00 ± 0.9 mm to 2.97 ± 0.95 mm C: from 2.03 ± 0.65 mm to 3.28 ± 1.14 mm	T: from 0.83 ± 0.26 mm to 1.49 ± 0.32 mm C: from 0.86 ± 0.29 mm to 1.57 ± 0.35 mm	MCAT in conjunction with either CM or CTG for MAGR is likely to show a relapse over a period of 9 years
RCT [29]	McGuire et al.	2022	CAF for single recessions	CAF + VCMX	CAF + CTG	SPLIT MOUTH 30/60	12	T: 63.2% C: 70.7%	T: NA C: NA	T: from 2.5 ± 1.25 mm to 3.3 ± 1.3 mm C: from 2.3 ± 0.88 mm to 3.6 ± 1.31 mm	T: from 158.37 ± 72.89 to 72.35 ± 38.40 mm^2^ C: from 189.40 ± 73.87 to 39.23 ± 30.92 mm^2^	VCMX + CAF root coverage was inferior to CTG + CAF but produced less morbidity
SR [17]	Halim et al.	2023	CAF	1. XDM 2. XDM 3. XDM 4. XDM 5. ADM	1. CTG 2. CTG 3. CTG 4. CTG 5. CTG	5 studies in total, 5 studies evaluated	>6	T: 70.3 C: 83.3 T: 24.3 ± 8.2 C: 48.7± 6.8 T: 70.7 C: 87.7 T: 51.9 C: 46.8 T: NA C: NA	T: 91.79 ± 10.1 C: 89.19 ± 16.3 T: 80.6 ± 23.7 C: 68.8± 23.4 T: NA C: NA T: 87.6 ± 15.1 C: 85.25 ± 14.9 T: NA C: NA	T: 0.85 ± 0.25 C: 0.81 ± 0.23 T: 0.8 ± 0.3 C: 0.8 ± 0.2 T: NA C: NA T: 0.69 ± 0.26 C: 0.61 ± 0.2 T: NA C: NA	T: 2.42 ± 1.29 C: 2.43 ± 1.12 T: 2.2 ± 1.3 C: 2.1± 1.6 T: 3.7 ± 1.10 C: 3.40 ± 1.2 T: 2.43 ± 1.4 C: 2.44 ± 1.3 T: 2.05 ± 0.78 C:1.90 ± 0.54	CTG is considered superior for gingival recession therapy. If it is contraindicated, the AADM and XDM might be considered as alternatives

ADM: allogeneic dermal matrix; C: control group; CAF: coronally advanced flap; CRC: complete root coverage; CTG: connective tissue graft; FGG: free gingival graft; mRC: mean root coverage; NA: not applicable; STT: soft tissue thickness; T: test group; TUN: tunnel technique; XDM: xenogeneic dermal matrix; CMX: xenogeneic collagen matrix; VCMX: volume-stable collagen matrix.

**Table 4:** Soft Tissue Substitutes vs. Free Gingival Graft/Connective Tissue Graft around Dental Implants.

Study Type	Authors	Year	Surgical Procedure	Test Group	Control Group	No. of Patients/ No. of Teeth or Implants	Follow-Up (Months)	CRC	mRC	KTW	STT	Conclusion
RCT [18]	Frizzera et al.	2018	STA (Immediate implant placement and provisionalization)	STA + CMX	STA + CTG	16/16	12	T: NA C: NA	T: NA C: NA	T: NA C: NA	T: from 0.98 to 3.04 mm C: from 1 to 2.11 mm	CTG avoided marginal peri-implant recession and provided greater thickness of the soft tissue at the implant facial aspect
RCT [30]	Thoma et al.	2020	STA before implant placement	STA + VCMX	STA + CTG	20/20	3 years	T: NA C: NA	T: NA C: NA	T: NA C: NA	T: from 3.0 to 3.5 mm C: from 3.0 to 3.3 mm	Both VCMX and CTGdemonstrated negligible differences, stable buccal tissue contour, esthetics, and STT slightly increased
SR [22]	Moraschini et al.	2022	CAF	1. CAF + CMX 2. CAF + CMX 3. CAF + CMX 4. CAF + CMX 5. CAF + CMX	1. CAF + CTG 2. CAF + CTG 3. CAF + CTG 4. CAF + CTG 5. CAF + CTG	11 studies in total, 5 studies evaluated	>6	T: NA C: NA	T: NA C: NA	1. XCM: 2.1 ± 1.2 mm CTG: 3.2 ± 0.8 mm 2. T: NA C: NA 3. XCM:1.7 ± 1.3 mm CTG:4.0 ± 1.1 mm 4. T: NA C: NA 5. XCM:6.51 ± 1.98 mm FGG: 7.76 ± 1.99 mm	1. XCM: 2.8 ± 0.7 mm CTG:3.1 ± 1.3 mm 2. XCM: 2.5 ± 1.3 mm CTG: 3.28 ± 1.7 mm 3. T: NA C: NA 4. XCM: 1.66 ± 0.01 mm CTG: 2.86 ± 0.01 mm 5. T: NA C: NA	CTG demonstrated best treatment ranking of probability results than CMX
RCT [20]	Lee et al.	2023	STA in conjunction with implant placement	STA + ADM	STA + CTG	30/30	12	ADM showed soft tissue margin 3–5 mm below the initial level	T: NA C: NA	Changes between the groups were not significantly different	T: from 1.34 ± 0.25 mm to 2.57 ± 0.3 mm C: from 1.24 ± 0.25 mm to 2.38 ± 0.32 mm	STA enhanced soft tissue thickness and maintained soft tissue contours but did not prevent peri-implant mucosal recession
RCT [31]	Thoma et al.	2023	STA after implant placement	STA + VCMX	STA + CTG	20/20	5 years	T: NA C: NA	T: NA C: NA	T: NA C: NA	T: from 3.0 to 3.0 mm C: from 3.0 to 3.3 mm	Both groups resulted in stable peri-implant tissues, favorable esthetics, and clinically negligible contour changes

ADM: allogeneic dermal matrix; C: control group; CAF: coronally advanced flap; CRC: complete root coverage; CTG: connective tissue graft; FGG: free gingival graft; mRC: mean root coverage; NA: not applicable; STA: soft tissues augmentation procedure; STT: soft tissue thickness; T: test group; CMX: xenogeneic collagen matrix; VCMX: volume-stable collagen matrix.

## 5. Discussion

Comparison of Soft Tissue Substitutes vs. No Treatment.

1. Allogeneic dermal matrix (ADM)

1.1. Soft tissue augmentation procedures around teeth

A meta-analysis by Chambrone et al. [4] (see Table 1) indicated no statistically significant difference between the coronally advanced flap (CAF) plus ADM graft and CAF alone concerning complete root coverage (CRC), recession reduction (RecRed), and keratinized tissue width (KTW) gain. It is crucial to note that these findings originated from two studies focusing on single recession defects with a 6-month follow-up [32,33].

These data are in disagreement with the only RCT study at a 12-month follow-up comparing ADM in the treatment of multiple gingival defects [34]. Inter-group differences were found to be statistically significant for RecRed, attachment gain, KTW and GT increase, and mean defect coverage in favor of the test group (*p* < 0.05).

1.2. Peri-implant soft tissue augmentation procedures

Only 1 RCT dealing with ADM vs. no treatment was found [20]. Results showed that the mean peri-implant mucosal thickness in the immediate implant with ADM was generally greater than in the control group at the 12-month visit (*p* = 0.063). However, it is interesting to note that thickened peri-implant mucosa did not significantly reduce peri-implant mucosal recession (Table 2).

2. Xenogeneic acellular dermal matrix (XDM)

Currently, there is no available evidence regarding the outcomes of using XDM in comparison to no graft for both soft tissue augmentation around teeth and peri-implant soft tissue augmentation procedures.

3. Bilayered collagen matrix (CMX)

3.1. Soft tissue augmentation procedures around teeth

A recent systematic review and network meta-analysis aimed to rank different biomaterials used in adjunct to CAF, based on their performance in root coverage for gingival recessions [35]. The authors concluded that all biomaterials (CTG, ADM, platelet concentrates, and CMX) had superior performance compared to CAF alone, for probing depth, keratinized tissue width, clinical attached level, and recession depth parameters.

These results confirmed data provided by a previous SR [21] and one RCT [23] whose clinical data are reported in Table 1.

Different data were reported in a 12-month randomized controlled trial comparing CMX + CAF to CAF in the treatment of single recession defects [36]. The CAF + CMX showed a higher STT gain (CAF, 0.1 ± 0.3 mm; CAF + CM, 0.6 ± 0.2 mm; *p* = 0.0001) and KTW gain (CAF, 0.3 ± 0.6 mm; CAF + CM, 0.9 ± 0.8 mm; *p* = 0.002) when compared with the CAF group. On the contrary, the estimated %RC did not present a significant difference between the groups (CAF, 70.3 ± 22%; CAF + CM, 69 ± 21.6%; *p* = 0.7) (Table 1).

3.2. Peri-implant soft tissue augmentation procedures

Limited evidence is currently available comparing the effect of applying a CMX with no soft tissue graft during implant placement (Table 2). A recent 12-month RCT was conducted to assess whether grafting the buccal peri-implant mucosa using either a CTG or CMX at implant placement in preserved alveolar ridges resulted in less mid-buccal mucosa recession compared to no grafting (NG) [19]. Of the patients, 90%, 75%, and 70% in the NG, CTG, and CMX groups, respectively, displayed more than 2 mm of keratinized mucosa. A 1- to 2-mm-wide zone of keratinized mucosa was seen in 5%, 15%, and 10% of patients in the NG, CTG, and CMX groups, respectively. In the CMX group, 5% of the patients had a keratinized mucosa of up to 1 mm. In the NG, CTG, and CMX groups, 5%, 10%, and 15%, respectively, of the patients showed no keratinized mucosa.

In another study [18], no significant differences were observed between the CMX graft and control group around implants in terms of STT, while Lee et al. [20] reported significant differences in terms of STT. No significant prevention of peri-implant mucosal recession was provided by the CMX group.

4. Volume-stable collagen matrix (VCMX)

Currently, there is no available evidence regarding the outcomes of using this newly introduced collagen matrix in comparison to no graft, for both soft tissue augmentation procedures around teeth and peri-implant soft tissue augmentation procedures alone.

Comparison of soft tissue substitutes vs. CTG/FGG.

1. Allogeneic dermal matrix (ADM)

1.1. Soft tissue augmentation procedures around teeth.

The literature examining the effectiveness of ADM (acellular dermal matrix) in root coverage procedures is extensive, showing a CRC range from 0% to 91% (Table 3). The data reported by Chambrone et al. [4] in their systematic review indicated that ADM yielded the most similar outcomes to the gold standard “connective tissue graft” (CTG).

A recent systematic review by de Carvalho Formiga et al. [16] comparing CTG with ADM in localized gingival recession defects reported similar CRC outcomes. Interestingly, when considering different thresholds of recession depth, CTG demonstrated superiority in 2 mm recessions, whereas it yielded comparable results in 3 mm recessions.

In contrast, a randomized controlled trial addressing multiple gingival recessions treated with the tunnel technique in combination with ADM or CTG revealed significant RecRed, increased gingival thickness (GT), and gains in clinical attachment level (CAL) in both groups nine months post-treatment [27]. The control group displayed more significant gains in KTW (CTG: 1.15 ± 1.16 mm vs. ADM: 0.21 ± 0.84 mm, *p* = 0.0003), increased GT (CTG: 0.94 ± 0.52 mm vs. ADM: 0.53 ± 0.41 mm, *p* = 0.002), and a higher percentage of mean root coverage (mRC) (CTG: 82.62 ± 16.30% vs. ADM: 72.72 ± 23.36%; *p* = 0.046), while RecRed and CAL gain did not significantly differ between the groups.

1.2. Peri-implant soft tissue augmentation procedures

Only one RCT [20] assessing tissue alterations in immediate implant sites with SCTG or ADM, compared to sites without soft tissue augmentation at 12 months, showed that STA with ADM at the immediate implant site did not maintain the peri-implant mucosal level better than STA with SCTG or no STA. In addition, it was reported a slight decrease in mean KMWs across all three groups compared to baseline. However, the changes in KMW were not significantly different among these groups (Table 4).

2. Xenogeneic acellular dermal matrix (XDM)

2.1. Soft tissue augmentation procedures around teeth

A recent systematic review aimed to compare long-term (≥1 year) root coverage outcomes between allogeneic and xenogeneic dermal matrices and the established gold standard, CTG [17] (Table 3). Despite a limited number of qualifying studies over the past decade (2014 to March 2023), only five findings meeting the inclusion criteria—with four specifically comparing XDM to CTG [26,37,38,39,40]—were primarily reported at a 12-month follow-up, except for Gurlek et al. [39], which extended to 18 months. The overall analysis of KTW across these studies indicated a mean difference of 0.26 mm (95% CI: −0.5 to 0.02). Additionally, a meta-analysis of mean root coverage (mRC) derived from three studies revealed a mean difference of 9.19% (95% CI: −13.95 to −4.43) [37,38,40]. Notably, both parameters favored CTG over dermal matrices.

In a similar vein, Santamaria et al. [36] conducted a study comparing clinical outcomes between XDM and CTG in conjunction with MCAF and cervical partial restoration for multiple gingival recessions. Their findings at a 1-year follow-up demonstrated a CRC of 50.7% for XDM and 72.9% for CTG, indicating a statistically significant disparity between the two groups (*p* < 0.001). Moreover, CTG exhibited more substantial increases in both KTW (CTG: 0.96 mm vs. XDM: 0.3 mm, *p* = 0.04) and gingival thickness (CTG: 0.9 mm vs. XDM: 0.3 mm, *p* < 0.001).

2.2. Peri-implant soft tissue augmentation procedures

Currently, there is no available evidence regarding the outcome of using XDM in comparison to CTG/FGG around an implant for soft tissue augmentation procedures.

3. Bilayered collagen matrix (CMX)

3.1. Soft tissue augmentation procedures around teeth

A recent meta-analysis [16] focusing on single recession defects calculated a weighted mean of −7.63 ± 5.43% for mean root coverage (mRC). This suggested a leaning toward CTG being more effective than CMX, though this trend lacked statistical significance.

This trend aligns with Tonetti et al.’s [26] findings for multiple recessions, yet contrasts with Aroca et al.’s study [24], which favored CTG (CMX: 73.2 ± 21.0% vs. CTG: 88.0 ± 20.9%; *p* = 0.021).

In a recent study [28] presenting the 9-year outcomes of a prior RCT by Aroca et al. [24], among the 16 participants from the 9-year follow-up, mRC decreased from 73.2% to 23.0% in the CMX group and from 88.0% to 39.7% in the CTG group. Interestingly, there were no significant differences between the groups after 9 years (*p* = 0.179). Notably, both the CMX and CTG groups showed increases in keratinized tissue width (KTW) and mucosal thickness (MTT), with no significant differences between them (*p* = 0.7197 for KTW; *p* = 0.8403 for MTT).

McGuire et al. [25] reported data at a 6–8-year follow-up comparing FGG and CMX, which showed a not inferior behavior of CMX (ΔRec = −0.07 ± 1.26 mm for CMX and −0.17 ± 0.78 mm for FGG, *p* = 0.710) (Table 3).

3.2. Peri-implant soft tissue augmentation procedures

In a recent network meta-analysis [22] comparing the augmentation of KMW between soft tissue substitutes and autogenous grafts, the findings indicated that FGG demonstrated the best treatment ranking of probability results, followed by connective tissue graft (CTG), acellular dermal matrix (ADM), and xenogeneic collagen matrix (XCM). Specifically, for the variables ‘mucosa thickness’ and ‘participant satisfaction with aesthetics’, the results showed the following ranking: CTG > ADM > XCM and XCM > ADM > CTG.

These data were confirmed by Frizzera et al. [18], who reported that the use of CTG avoided marginal peri-implant recession (*p* < 0.05) and provided better alveolar ridge contour (*p* < 0.01) and greater thickness (*p* < 0.05) of the soft tissue at the facial aspect than CMX-treated sites (Table 4).

4. Volume-stable collagen matrix (VCMX)

4.1. Soft tissue augmentation procedures around teeth

Currently, there is limited available evidence regarding the root coverage outcomes using this newly introduced collagen matrix in comparison to those achieved with CTG.

Findings from a randomized controlled trial [29] investigating single gingival recessions revealed that at the 1-year follow-up, the mean root coverage was 63.2 ± 31.56% for the VCMX group and 84.49 ± 19.98% for the CTG group. There was a statistically significant difference between the two groups (*p* < 0.0001). In terms of changes in keratinized tissue width (KTW) at the 1-year follow-up, the CTG group exhibited a shift from 2.3 ± 0.88 mm to 3.6 ± 1.31 mm, while the VCMX group shifted from 2.5 ± 1.25 mm to 3.3 ± 1.3 mm (Table 3).

4.2. Peri-implant soft tissue augmentation procedures

Likewise, there is limited evidence available when comparing outcomes following soft tissue augmentation procedures using VCMX and CTG. Only two randomized controlled trials, encompassing one patient cohort, reported results of this comparison at 3- and 5-years [30,31]. At the 5-year follow-up, implant sites augmented with VCMX displayed a slight, though not statistically significant, increase in KMW from 3 years (2.5 mm) to 5 years (3.1 mm). In contrast, implants treated with CTG exhibited a mean KMW of 3.2 mm at the 3-year follow-up and 3.3 mm at the 5-year visit. However, the authors did not observe any significant differences in KMW between VCMX and CTG. Regarding volumetric changes, both groups showed clinically negligible alterations from baseline to 5 years (VCMX: from 2.7 mm to 3.2 mm; CTG: from 3.2 mm to 3.4 mm) (Table 4).

## 6. Conclusions

Based on the available evidence, questions remain about the alternative use of soft tissue substitutes for conventional grafting procedures using free gingival grafts or connective tissue grafts around teeth and implants.

In particular, around *teeth*, all biomaterials show superior performance compared to CAF alone for treating gingival recessions. However, when compared to CTG, ADM yields the most similar outcomes to the gold standard (CTG), even though in multiple recessions CTG continues to be considered the most favorable approach. The use of STSs in combination with an apically positioned flap resulted in significantly less gain in KTW compared to what was achieved with FGG and APF.

Around *dental implants*, free gingival grafts were deemed more effective than soft tissue substitutes in enhancing keratinized mucosa width.

## Data Availability

All data are reported in the text.
